# Antibody Response to mRNA Vaccines against SARS-CoV-2 with Chronic Kidney Disease, Hemodialysis, and after Kidney Transplantation

**DOI:** 10.3390/jcm11010148

**Published:** 2021-12-28

**Authors:** Lukas Buchwinkler, Claire Anne Solagna, Janosch Messner, Markus Pirklbauer, Michael Rudnicki, Gert Mayer, Julia Kerschbaum

**Affiliations:** Department of Internal Medicine IV—Nephrology and Hypertension, Medical University Innsbruck, Anichstrasse 35, 6020 Innsbruck, Austria; lukas.buchwinkler@i-med.ac.at (L.B.); claire.solagna@student.i-med.ac.at (C.A.S.); janosch.messner@student.i-med.ac.at (J.M.); markus.pirklbauer@i-med.ac.at (M.P.); michael.rudnicki@i-med.ac.at (M.R.); gert.mayer@i-med.ac.at (G.M.)

**Keywords:** SARS-CoV-2, COVID-19, mRNA vaccines, kidney transplantation, hemodialysis, chronic kidney disease

## Abstract

Most trials on mRNA vaccines against SARS-CoV-2 did not include patients with chronic kidney disease (CKD), hemodialysis (HD) patients, or kidney transplant recipients (KTR). However, those patients have a higher risk for a severe course of COVID-19 disease and mortality. Available literature has demonstrated a reduced efficacy of mRNA vaccines in HD patients and KTR, while data on CKD patients is scarce. Additionally, factors associated with non-response are poorly understood and not well characterized. We assessed antibody (AB) response (*n* = 582, 160 CKD patients, 206 patients on HD, 216 KTR) after the administration of two doses of a mRNA-vaccine with either BNT162b2 or mRNA-1273. AB measurements were carried out after a median of 91 days after first vaccinations, demonstrating non-response in 12.5% of CKD patients, 12.1% of HD patients, and 50% of KTR. AB titers were significantly higher in CKD patients than in HD patients or KTR. Factors associated with non-response were treated with rituximab in CKD patients, the use of calcineurin inhibitors in HD patients and older age, and the use of BNT162b2, mycophenolic acid, or glucocorticoids and lower hemoglobin levels in KTR. This study contributes to the understanding of the extent and conditions that predispose for non-response in patients with impaired kidney function.

## 1. Introduction

To combat the ongoing COVID-19 pandemic, significant effort has been undertaken to develop highly effective and safe vaccines against SARS-CoV-2 (severe acute respiratory syndrome coronavirus 2). Two of the most promising vaccines are BNT162b2 by Pfizer and mRNA-1273 by Moderna. However, the respective authorization trials as well as most follow-up trials on these mRNA vaccines did not include patients with chronic kidney disease (CKD), on hemodialysis (HD), or after kidney transplantation (KT). Moreover, immunosuppressive treatment was an exclusion criterion in the original registration studies [1,2]. Several publications have already indicated a reduced humoral immune response in HD [3,4,5] and kidney transplant recipients (KTR) [6,7,8], while data in CKD patients with or without immunosuppressive treatment is scarce. Factors/conditions discussed to predispose non-response and a severe COVID-19 course in these patients are advanced age, comorbidities, the uremic environment in HD, and immunosuppressive medication in KTR. Factors that are associated with non-response in the existing literature include age [9,10], diabetes [11], lower hemoglobin [9], lower eGFR (per mL/min/1.73 m^2^) [12], longer HD vintage [13], BNT162b2 instead of mRNA-1273 [14,15,16,17], treatment with antimetabolites [18] or belatacept [19,20], treatment with rituximab [21] or high dose cortisone in the last year [9], and triple immunosuppression (calcineurin inhibitor plus antimetabolite plus steroid) [9]. The extent and impact of the abovementioned factors are poorly understood, and possible mitigation strategies are lacking.

Since mortality in SARS-CoV-2-infected kidney transplant recipients [22], hemodialysis patients [23], and CKD patients [24] is high, further understanding of the efficacy of the SARS-CoV-2 vaccination and risk factors for non-response in this highly vulnerable population is urgently needed. Safe and effective vaccination strategies remain especially important due to the fact that, as of date, effective treatments for severe COVID-19 disease are rarely available. Additionally, most of these patients have difficulties to socially distance due to either needing regular hemodialysis, medical appointments, or care through relatives or nursing personnel.

## 2. Materials and Methods

Between February and April 2021, CKD patients, HD patients, and KT recipients received two doses of mRNA-vaccination based on the recommendations of the Austrian National Advisory Committee on immunization practices. Patients were treated in Tyrolean dialysis centers or by the Department of Internal Medicine IV at the University Hospital Innsbuck. Data were analyzed retrospectively; inclusion in this study did not play any role in immunization practices. Routine laboratory parameters and clinical data were assessed in May and June 2021 through electronic patient records.

Antibody (AB) titers were measured by Abbott SARS-CoV-2 IgG II Quant Assay in 40% of each group and by Liaison^®^ SARS-CoV-2 S1/S2 IgG in 60% of each group based on availability in our laboratory between January and August 2021. Limits for non-response were <7 BAU/mL and <13 AU/mL, respectively, as defined by the manufacturer. Measurements with the Liaison assay were carried out and analyzed before the conversion of the assay to BAU/mL, hence the unit AU/mL is used (the now defined cut-off of 33.8 BAU/mL is equivalent to 13 AU/mL, conversion factor 2.6).

Inclusion criteria comprised either CKD (predominantly CKD stage IV and V), HD or living with a functioning kidney graft as underlying condition, completed prime-booster vaccination with a mRNA vaccine approximately 4 weeks apart, and an available measurement of AB titers with at least one of the above-mentioned assays 60 to 120 days after first vaccine dose. Exclusion criteria included age under 18 years at the time of first vaccination and a history of PCR-proven SARS-CoV-2 infection or the detection of nucleocapsid AB.

BNT162b2 was used in 58% of CKD patients, in 96% of HD patients, and in 49% of KTR; the remainder received mRNA-1273. Both doses were administered approximately 4 weeks apart (median 28 days, 25% and 75% percentiles were 21 and 29 days). AB measurement was carried out after a median of 91 days after the first vaccination.

Statistical analysis was performed with IBM SPSS Statistics. Non-parametric tests were used for the comparison of continuous data, and logistic regression served for assessment of risk factors for non-response. All factors showing a univariate association with a *p*-value < 0.100 were entered in the final multivariate model.

This analysis was approved by the Institutional Review Board of the Medical University Innsbruck (ECS 1280/2021).

## 3. Results

The initial cohort consisted of 871 patients with either CKD, treatment with HD or after kidney transplantation. Sixty-two patients were excluded due to a history of SARS-CoV-2 infection before the first or the second dose, and 17 patients were excluded due to nucleocapsid AB detection before the first or second dose without a clinically apparent history of infection. Nineteen patients had no second dose of vaccination. From the remaining 773 patients, in 191 patients, antibodies were not taken between 60 to 120 days after the first vaccination. Hence, in the final cohort, 582 patients were included: 160 patients with CKD, 206 patients on HD, and 216 patients after kidney transplantation. Table 1 displays baseline characteristics of the final cohort. 

The median age was 63.1, 69.5, and 59.9 years, respectively, in CKD patients, HD patients, and KTR. The median eGFR was 29.9 mL/min/1.73 m^2^ in CKD patients and 49.2 mL/min/1.73 m^2^ in KTR. Details on immunosuppressive treatment and comorbidities are also given in Table 1. The median time between the first vaccination and AB measurement was 91 days in all groups. Table 2 shows the type of vaccine used and the types and results of antibody measurement. Rates of non-response were 12.5%, 12.1%, and 50.0% in CKD patients, HD patients, and KTR.

**Figure 1:**
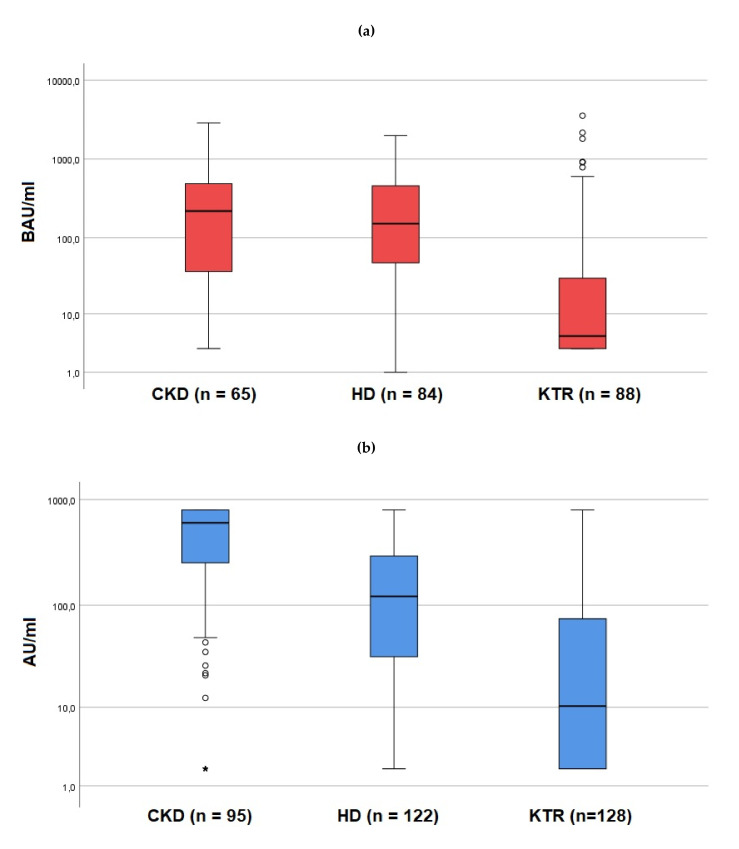
(**a**,**b**): Levels of antibody titers in CKD patients, HD patients, and KTR. (**a**) shows the results of the Abbott SARS-CoV-2 IgG II Quant Assay test (median, 25, and 75% percentile for CKD: 230.3 (48.3–497.6), for HD: 151.6 (47.7–458.4), for KTR: 4.8 (3.0–30.2); (**b**) shows the results of the Liaison^®^ SARS-CoV-2 S1/S2 IgG test (median, 25, and 75% percentile for CKD: 602.0 (252.5–800.0), for HD: 121.5 (32.0–293.0), for KTR: 10.3 (1.9–74.3). Logarithmic scale. Circles represent statistical outliers between 1.5 to 3.0 times of the interquartile range (IQR), asterisks represent statistical outliers more than 3 times of the IQR.

Levels of antibody titers were significantly higher in CKD patients (Abbott: 230.3 BAU/mL [48.3–497.6]; Liaison: 602.0 AU/mL [252.5–800.0]) than in HD patients (Abbott: 151.6 BAU/mL [47.7–458.4]; Liaison: 121.5 AU/mL [32.0–293.0]); (*p* < 0.001) and in KTR (Abbott: 4.75 BAU/mL [3.0–30.2]; Liaison: 10.3 AU/mL [1.9–74.3]); (all *p* < 0.001, Figure 1). 

In a multivariate analysis, risk factors for non-response were examined. In CKD, a significant risk factor for non-response was treatment with rituximab (OR 27.2, 95% CI 5.12–144.63, *p* < 0.001) before vaccination (Table 3). The rate of non-response was 53.3% in CKD patients treated with rituximab (*n* = 30) versus 3.1% in those without rituximab treatment (*n* = 130). Longer temporal distance of rituximab application to first vaccination significantly reduced the risk for non-response (OR 0.98, 0.96–1.00, *p* = 0.020 per day).

In HD patients with renal transplant in situ (*n* = 39), the use of calcineurin inhibitors was significantly associated with non-response (OR 14.85, 2.68–82.43, *p* = 0.002); no other risk factors could be identified in HD patients (Table 4).

In KTR, higher age (OR 1.06, 1.03–1.09, *p* < 0.001 per year) was significantly associated with non-response (Table 5). The use of mycophenolic acid (OR 6.61, 2.31–18.86, *p* < 0.001) or glucocorticoids (OR 4.95, 1.48–16.57, *p* = 0.010) was also significantly associated with non-response, whereas vaccination with mRNA-1273 (OR 0.41, 0.20–0.83, *p* = 0.014) and higher levels of hemoglobin (OR 0.97, 0.95–0.99, *p* < 0.001 per g/L significantly reduced the risk for non-response. Treatment with belatacept was not significantly associated with non-response, possibly due to a low patient number (*n* = 14).

Presence of diabetes or the underlying primary renal disease (diabetic nephropathy, vascular/hypertensive nephropathy, glomerulonephritis, other, unknown) did not predict non-response in any of the groups. In addition, the presence of cardiovascular or cerebrovascular disease were not associated with non-response. Further non-significant factors were treatment with RAAS inhibitors and the number of classes of antihypertensive drugs.

## 4. Discussion

This is one of the first studies showing a direct comparison of SARS-CoV-2 antibody titers in CKD patients with and without immunosuppressive treatment, HD patients, and KTR. We found levels of antibody titers to be significantly higher in CKD patients than in HD patients and KTR. Interestingly, primary non-response in CKD and HD patients was approximately the same (12.5% vs. 12.1%). The non-response rate in HD is in line with recent literature [25]. It has been demonstrated that immunosuppressive medication after transplantation impairs immune response to (not only SARS-CoV-2) vaccines [26] and influences immunization practices of listed HD patients [27]. Our data also suggests a possible advantage of vaccination before the initiation of renal replacement therapy (RRT).

The low AB titers in HD patients and KTR might indicate the need for a scheduled booster regimen to enhance immune response in those patients. In particular, the very low titers in KTR are alarming, not only regarding the potential of vaccines to protect against COVID-19 disease, but also the longevity of the supposed protection. Several trials have already reported on immune responses to a third vaccine dose in KTR non-responders [28,29,30] and weak responders [31], as well as HD patients [32,33]. Approximately 50% of KTR developed a positive AB response after a third dose. A fourth dose seems to have a similar efficacy [34]. This raises the question of the efficiency of available vaccines (or vaccination strategies) to grant adequate protection from COVID-19 in KTR. In the case of continuous non-/or very low-response, basic hygiene measures, social distancing, and especially a high vaccination coverage of care providers, relatives, and the general population remain highly important. To the best of our knowledge, no systematic analysis of efficacy of a third vaccine dose in CKD patients exists.

Recent publications demonstrated a 100% percent development of AB after vaccination in CKD patients [35,36]. Our finding of a response rate of 87.5% can be partially explained by the fact that, in contrast to these studies, we included CKD patients with immunosuppressive medication. In our cohort, 19% of CKD patients received rituximab treatment, while six patients received a high dose glucocorticoid treatment (>1 mg/kg body weight) during the last year. 

A limitation of our study is the fact that we did not assess cellular immune responses. While it is reasonable to assume that immunosuppressive medication impairs cellular immune response to a similar extent than humoral response (with the exception of rituximab), some studies exist that demonstrated a significant T-cell immunity in cases of humoral non-response [37,38]. The extent to which S1-AB titer correlates with protection against infection and severe COVID-19 courses is incompletely understood. An interesting point was raised in recent publications: while the development of positive AB titers seems to correlate reasonably well with neutralizing antibodies [39] and neutralizing capacity of patient plasma [40], 10% of seropositive KTRs did not develop neutralizing capacity [39]. Comparison and interpretation of cellular assays is difficult due to the heterogeneity of employed analyses. While the analysis of S1-AB assays might not be able to inform on the whole immunologic picture, an inexpensive tool, easy to deploy and compare, is needed to combat the ongoing pandemic and inform on sufficient or inadequate protection. The lack of follow-up is a limitation of our study; “real life” data on break-through infections and COVID-19 infection courses of vaccinated immunosuppressed patients are urgently needed. 

Furthermore, specific immunosuppressive agents (such as rituximab in CKD patients, calcineurin inhibitors in HD patients with transplant in situ, and mycophenolic acid and glucocorticoids in KTR) might influence immune response, suggesting a possible need for a change in immunosuppressive therapy ahead of vaccination. While changes to immunosuppression in KTR should be handled with caution, in HD patients temporarily pausing immunosuppressive medication prescribed to preserve residual kidney function seems feasible, though it is unclear if pausing immunosuppression is sufficient to restore an adequate vaccine response. Our data also suggests aiming for a temporal distance of vaccination to the last rituximab administration in CKD patients, which is in line with current literature [41]. While induction treatment with rituximab, for example in ANCA-associated vasculitis, usually cannot be delayed, postponing COVID-19 vaccination until the reconstitution of CD19+ B-cells, a transient switch to other immunosuppressive treatments (such as azathioprine) or a delay of rituximab treatment in stable patients may be warranted [42]. In any case, a careful benefit-risk analysis by the providing physician is needed in such instances.

In line with the available literature, our data favours the usage of mRNA-1273 over BNT162b2 in KTR [16,17] as well as HD [14,15] to ensure optimal response. As of to date, no such recommendation has found entry in international, national or scientific guidelines on vaccination strategies.

The data of our study were collected at a time where SARS-CoV-2 variants of concern (Delta, Omicron) did not play a major epidemiological role in Austria.

In general, the vaccination of household members and other close contacts is essential and has been shown to reduce the transmission rate [43].

In summary, our study expands the knowledge of AB response to mRNA-based SARS-CoV-2 vaccinations in different groups of patients with impaired kidney function. Patients with CKD had a significantly better immune response than patients on HD or KTR, while still over 10% of CKD patients did not mount a detectable immune response. Clinical studies on antibody levels as well as cellular immune responses, duration of protection, and break through infections in those patients are clearly needed to inform on future immunization strategies and optimal treatment.

## Figures and Tables

**Table 1:** Data is displayed as median, 25%, and 75% percentiles. Numeric data is displayed as number of participants (*n*) and percentage (%) where appropriate (sums do not add up to 100% due to rounding).

	CKD (*n* = 160)	HD (*n* = 206)	KTR (*n* = 216)
Age (years)	63.1 (53.5–74.5)	69.5 (57.5–78.6)	59.9 (50.7–68.5)
Female (*n*, %)	66 (41)	66 (32)	69 (32)
BMI (kg/m^2^)	25.6 (23.3–29.3)	24.9 (22.2–28.4)	24.8 (21.7–27.8)
Primary renal disease (*n*, %)			
Diabetic nephropathy	14 (9)	45 (22)	28 (13)
Glomerulonephritis	64 (40)	35 (17)	71 (33)
Other	49 (31)	55 (27)	77 (36)
Unknown	3 (2)	23 (11)	11 (5)
Vascular nephropathy	30 (19)	48 (23)	29 (13)
Comorbidities (*n*)			
Cardiovascular disease	45	98	92
Cerebrovascular disease	16	33	34
Active or former malignancy	13	35	53
Diabetes mellitus	36	77	79
Comedication (*n*, %)			
Treatment with RAAS inhibitors	83 (52)	62 (30)	93 (43)
High-dose glucocorticoid treatment during last year (≥1 mg/kg)	6 (4)	4 (2)	16 (7)
Tacrolimus	-	7 (3)	152 (70)
Cyclosporine A	-	1 (0.5)	38 (18)
Azathioprine	-	0	33 (15)
Mycophenolic acid	-	2 (1)	148 (69)
Belatacept	-	0	14 (6)
Glucocorticoids	-	8 (4)	178 (82)
mTor inhibitors	-	0	11 (5)
Rituximab	30 (19)	-	-
Time between 1st vaccination and rituximab (days)	242 (127–324)	2 (1–4)	2 (1–3)
Classes of antihypertensive drugs (*n*)	2 (1–3)		
Laboratory values			
Albumin (g/dL)	4.0 (3.7–4.3)	3.8 (3.4–4.1)	
Hemoglobin (g/dL)	132 (117–141)	111 (105–120)	4.1 (3.9–4.4)
C-reactive protein (mg/dL)	0.20 (0.09–0.46)	0.33 (0.16–0.95)	134 (122–146)
eGFR (mL/min/1.73 m^2^)	29.9 (19.8–49.9)	-	0.19 (0.08–0.40)

**Table 2:** Type of vaccine and antibody measurement in CKD and HD patients, and KTR. Continuous data is displayed as median, 25%, and 75% percentiles. Numeric data is displayed as number of participants (*n*) and percentage (%) where appropriate (sums do not add up to 100% due to rounding).

	CKD (*n* = 160)	HD (*n* = 206)	KTR (*n* = 216)
Type of antibody measurement (*n*, %)			
Abbott SARS-CoV-2 IgG II Quant Assay	65 (40.6)	84 (40.8)	88 (40.7)
Liaison^®^ SARS-CoV-2 S1/S2 IgG	95 (59.4)	122 (59.2)	128 (59.3)
Vaccine (*n*, %)			
BNT162b2	93 (58.1)	198 (96.1)	106 (49.1)
mRNA-1273	67 (41.9)	8 (3.9)	110 (50.9)
Results of antibody measurement			
Abbott SARS-CoV-2 IgG II Quant Assay titer (BAU/mL)	230.3 (48.3–497.6)	151.6 (47.7–458.4)	4.75 (3.0–30.2)
Liaison^®^ SARS-CoV-2 S1/S2 IgG titer (AU/mL)	602.0 (252.5–800.0)	121.5 (32.0–293.0)	10.3 (1.9–74.3)
Non-response (*n*, %)	20 (12.5)	25 (12.1)	108 (50.0)
Time between 1st vaccination and AB measurement (days)	91 (90–96)	91 (88–94)	91 (90–95)

**Table 3:** Multivariate analysis of risk factors for non-response in CKD patients. OR odds ratio, CI confidence interval.

	OR (95% CI)	*p*-Value
Glomerulonephritis	1.53 (0.24–9.75)	0.651
Rituximab treatment	27.20 (5.12–144.63)	<0.001
Days between 1st vaccination and Rituximab (per day)	0.98 (0.96–1.00)	0.020

**Table 4:** Multivariate analysis of risk factors for non-response in HD patients. OR odds ratio, CI confidence interval.

	OR (95% CI)	*p*-Value
Glomerulonephritis	2.87 (0.95–8.70)	0.062
High-dose glucocorticoid treatment during last year (≥1 mg/kg)	6.36 (0.67–60.44)	0.107
Treatment with tacrolimus or cyclosporine A	14.85 (2.68–82.43)	0.002
Glucocorticoids	1.90 (0.25–14.49)	0.537
Hemoglobin (per g/dL)	0.97 (0.95–1.01)	0.098

**Table 5:** Multivariate analysis of risk factors for non-response in KTR. OR odds ratio, CI confidence interval, KT kidney transplantation.

	OR (95% CI)	*p*-Value
Age (per year)	1.06 (1.03–1.09)	<0.001
Type of vaccination		
BNT162b2	Ref.	-
mRNA-1273	0.41 (0.20-0.83)	0.014
Cerebrovascular disease	3.11 (0.99–9.76)	0.052
CMV reactivation during the last 6 months	1.22 (0.47–3.18)	0.681
Basis of immunosuppression		
Antimetabolite and/or steroids	Ref.	-
Cyclosporine A	0.89 (0.06–14.23)	0.934
Tacrolimus	1.59 (0.11–22.38)	0.730
mTOR inhibitors	0.31 (0.01–10.42)	0.512
Belatacept	11.01 (0.45–269.68)	0.142
Antimetabolites		
No antimetabolites	Ref.	-
Azathioprine	0.73 (0.18–2.92)	0.652
Mycophenolic acid	6.61 (2.31–18.86)	<0.001
Glucocorticoids	4.95 (1.48–16.57)	0.010
Hemoglobin (per g/dL)	0.97 (0.95–0.99)	<0.001
Time since last KT (per year)	0.99 (0.94–1.04)	0.660

## Data Availability

The data presented in this study are available on request from the corresponding author. The data are not publicly available due to data protection.
